# Nordic walking training attenuation of oxidative stress in association with a drop in body iron stores in elderly women

**DOI:** 10.1007/s10522-017-9681-0

**Published:** 2017-02-22

**Authors:** Jakub Kortas, Agnieszka Kuchta, Krzysztof Prusik, Katarzyna Prusik, Ewa Ziemann, Sandra Labudda, Agnieszka Ćwiklińska, Ewa Wieczorek, Maciej Jankowski, Jedrzej Antosiewicz

**Affiliations:** 1 Department of Recreation and Qualify Tourism, Gdansk University of Physical Education and Sport, Kazimierza Górskiego 1, 80-336 Gdansk, Poland; 2 Department of Clinical Chemistry, Medical University of Gdansk, Gdansk, Poland; 3 Department of Biomedical Basis of Health, Gdansk University of Physical Education and Sport, Gdansk, Poland; 4 Department of Physiology and Pharmacology, Gdansk University of Physical Education and Sport, Gdansk, Poland; 5 Department of Biochemistry, Gdansk University of Physical Education and Sport, Gdansk, Poland

**Keywords:** Exercise, Physical activity, Lipid peroxidation, Healthy lifestyle, Iron metabolism

## Abstract

Excess body iron accumulation and oxidative stress has been associated with ageing. Regular exercise has been shown to reduce oxidative stress and induce some changes in iron metabolism. However, the effects of exercise on both of these parameters have been poorly investigated. In our study, 35 elderly women participated in 12 weeks of Nordic walking (NW) training (three times a week). We demonstrated that the training caused a significant reduction in malondialdehyde advanced oxidation protein products—markers of oxidative stress but had no effects on paraoxonase 1 activity. These changes were associated with the decrease of blood ferritin (99.4 ± 62.7 vs. 81.4 ± 61.7 ng/ml p < 0.05). Measurement of physical fitness revealed that the training caused a significant improvement in performance and a negative correlation between the blood ferritin and endurance test was recorded (r = −0.34, p = 0.03). In addition, a significant correlation between blood ferritin and fasting glucose level was noted. The training induced a rise of HDL cholesterol from 70.8 ± 19.3–75.3 ± 21.1, p < 0.05, whereas other lipid parameters remained unchanged. In conclusion, NW training reduced body iron stores and it was associated with lower oxidative stress and better endurance.

## Introduction

Currently 11% of the world population are people above 60 years old, of which 14% are over 80 years old. Demographic prognoses indicate that in 2050 the number of elderly people will be up threefold to about two billion. Regular physical activity is related with successful healthy aging. Many studies suggest that participating in regular physical activity has significant benefits for health (Alburquerque-Sendin et al. 2012; Kuzmin et al. 2015; Mathieson and Lin 2014; Tschentscher et al. 2013), including improved treatment of many diseases (Hanuszkiewicz et al. 2014). Thus, health training plays an important role not only in improving the quality of life among the elderly (Halaweh et al. 2015), but also in supporting their independent functioning (Shinkai et al. 2016), which is a major social and economic problem of this demographic. We are still far from a complete understanding of the mechanisms responsible for healthy effects of exercise. Skeletal muscle is known to produce and secrete many myokines, which can modify the metabolism of other organs such as the liver, adipose tissue, the brain and some others (Pedersen 2011). Physical activity is also associated with increased consumption of oxygen in the muscles, which leads to an increased production of reactive oxygen species (ROS). Reactive oxygen species on the one hand can cause damage of cell structures, on the other hand they are important signalling molecules, induce adaptation process (Radak et al. 2005) and influence myokines production (Ost et al. 2016). The trace amounts of ‘free’ iron, also known as labile iron pool (LIP), can change the quantity and quality of produced ROS (Kruszewski 2003). Iron catalyses production of a highly toxic hydroxyl radical via the Fenton/Haber–Weiss reaction cycle. Therefore, changes in LIP level significantly influence ROS formation (Kruszewski 2003). One of the major sources of LIP is iron stored in ferritin, which is present in every human cell. During stress conditions, part of the iron is released from the ferritin and iron-dependent ROS formation has been demonstrated (Borkowska et al. 2011). Based on this and other studies it has become clear that stored iron is not safe, as suggested previously, and can determine free radicals-mediated damage (Fernandez-Real et al. 2002; Sullivan 2004). In addition, several studies have demonstrated that the higher the iron stores, the higher the risk of several morbidities including cancer, heart attack, diabetes and other (Jehn et al. 2004).

Different kind of exercise has been shown to the rise of blood level of hepcidin a hormone regulating iron metabolism (Skarpanska-Stejnborn et al. 2014). Hepcidin, by blocking ferroprotin in enterocytes, inhibits the dietary iron absorption (Ganz and Nemeth 2011); possibly, this might be the mechanism responsible for lower body iron stores in people, who are physically active (Kortas et al. 2015; Lakka et al. 1994; Vdovenko et al. 2015). The rise of hepcidin recorded after exercise might also be a kind of defence against to the inflammation induced by elevated physical workload.

Oxidative stress can have a significant role in the pathogenesis of many diseases (Phaniendra et al. 2015) and there is a correlation between age and the level of oxidative stress (Bouzid et al. 2015).

In this paper we hypothesise that regular Nordic Walking exercise will reduce oxidative stress by lowering body iron stores in elderly subjects. We report that 12 weeks of NW training significantly diminish oxidative stress and that this was accompanied by lower blood ferritin and iron concentration. These changes were also accompanied by an improvement of physical fitness and blood lipids profile.

## Materials and methods

A group of 35 elderly women participated in the study. All of them were older than 60 years (68 ± 5.12 years old). All the subjects underwent a medical check-up prior to the experiment. Those with uncontrolled hypertension (diastolic blood pressure over 100 mmHg), a history of cardiac arrhythmia, cardio-respiratory disorders, and orthopaedic problems were excluded from the study. The analysis and training programme were completed at the Gdansk University of Physical Education and Sport. It was recommended that the volunteers did not change their diet throughout the experiment.

### Ethics statement

The examination was officially approved by the Bioethical Committee of the Regional Medical Society in Gdansk (KB-26/14) according to the Declaration of Helsinki. Before commencing the training and testing, the subjects received a verbal description of the experiment. Written informed consent was signed by all the participants. Ethics approval was obtained for the referral of participants to their family physician upon detection of any abnormal pathology results and review by the study medical officer.

### Blood analysis

At baseline and one day directly after the 12-week training programme, blood samples were obtained between 7 and 8 a.m. following an overnight fast. The serum were separated by centrifugation at 1000×g for 15 min and stored at −80 °C pending analysis. Total cholesterol (TC) and triacylglycerols (TAG) were measured in the serum using standard enzymatic colorimetric tests. High-density lipoprotein cholesterol (HDL-C) was determined following precipitation of apolipoprotein B containing lipoproteins; LDL cholesterol level (LDL-C) was calculated using the Friedewald formula.

Paraoxonase (PONase) and arylesterase (AREase) activity of paraoxonase -1 were measured in the serum based on paraoxon and phenyl acetate hydrolysis, respectively, according to the procedure described earlier (MacKness et al. 2000; Nakanishi et al. 2003). Lipid peroxide formation was estimated as malondialdehyde (MDA) concentration with fluorescence spectroscopy according to the methods of (Yokode et al. 1988). Advanced oxidative protein products (AOPP) determination was based on spectrophotometric detection according to (Witko-Sarsat et al. 1996) with modification of (Anderstam et al. 2008) and expressed as chloramine-T equivalents. The antioxidant capacity of serum was measured via the ferric reducing ability potential (FRAP) assay as described by (Benzie and Strain 1996) using trolox as a standard.

### Measurements of physical fitness

A battery of field tests specifically developed for older adults were used to assess the components of functional fitness. These tests require very little time or equipment and are designed to be conducted in community settings. In accordance with Rikli and Jones (Jones et al. 1999; Rikli and Jones 2013), we used the following tests at the beginning of the study and after 12 weeks. The Senior Fitness Test (SFT) consists of six items. The SFT items are as follows: (1) 30-s chair stand; (2) arm curl; (3) chair sit-and-reach; (4) back scratch; (5) 2-min step; and (6) 8-foot up-and-go. The tests were performed in this order with 1 min of rest between them. Before each test, the evaluator demonstrated the exercise and the participant carried out an attempt at familiarisation, except for the walking test which the subjects performed only once.

### Exercise protocol

The same group of research assistants and coaches supervised all the training sessions. The experimental group completed 12 weeks’ of mesocycle exercise, which included 35 individuals, divided into three microcycles. The participants met three times a week, 1 h after eating a light breakfast (10-min warm-up, 45–55-min NW, and 10-min cool-down) and performed the main session of NW training at 60–70% intensity of the maximal HR. Each training unit was recorded by Garmin Forerunner 405 with built-in GPS. During the applied training programme all of the women covered 107 km 300 m. Once a week, each participant received a sport-tester device type used for current cardiovascular control.

The main task of the first microcycle (six training sessions) was to adapt the body to undertake regular physical activity and to learn the specialist exercise, supporting the correct technique of walking with sticks. The main aim was also to improve chest mobility and increase flexibility of the arms and shoulders. Educating the women in the correct hold and set of the sticks during walking was also the purpose of the training. The second microcycle included 24 training units and was an essential part of the programme. The aim of this part was to improve endurance. Implementation of this part was characterised by a gradual increase in volume (expressed in walking kilometres), which was inherent to increasing the training intensity.

The closing microcycle (six training units) was an attempt to raise the level of endurance by intensifying activities and walking at the fastest possible pace.

For safety reasons the designated operating ranges were not the maximum physiological capabilities, but the actual willingness to undertake training and mobilisation for physical activity in this period of life. Elderly people have a high barrier to participation in physical training, often associated with their thinking that intensive training may lead to illness. Accordingly, the last mesocycle training was a year of setting new boundaries, mainly in the sphere of psychological activities for the participants. The main objective of the programme—the formation of endurance—was realised by achieving the maximum intensity of the walking test.

### Statistical analysis

All statistical analyses were performed using Statistica 12.0 software. All values are expressed as mean ± standard deviation (SD). The Wilcoxon signed-rank test was applied to evaluate changes in anthropometric and biochemical parameters. The relation-ships between variables were evaluated using a Pearson cor-relation coefficient. Statistical significance was set at p < 0.05.

## Results

### General outcomes

The baseline clinical characteristics of the participants are summarised in Table 1. Body mass index 95% confidential interval for mean ranged from 24.49 to 27.56. The 12 weeks of Nordic walking training caused no significant changes in body composition beside the free fat mass, where we observed a significant increase.

**Table 1:** Anthropometric characteristics of participants (n = 35)

	Baseline	After 12 weeks of training	p
Weight [kg]	68.44 ± 10.04	68.94 ± 9.4	0.06
BMI [kg·m^−2^]	26.23 ± 3.83	26.43 ± 3.56	0.06
Fat [kg]	23.97 ± 7.5	24.26 ± 7.31	0.56
Fat [%]	34.28 ± 6.62	34.61 ± 7.46	0.94
FFM [kg]	44.47 ± 4.22	44.69 ± 5.21	**0.04**
TBW [kg]	32.63 ± 3.1	32.79 ± 3.78	0.06

Values are means (±SD)

*BMI* body mass index, *Fat* fat mass, *Fat* % percentage of body fat, *FFM* free fat mass, *TBW* total body water

### Oxidative stress balance

A statistically significant decreasing of malondialdehyde level (mean change = −21.52; 95% CI −36.64 to −6.39) and concentration of advanced oxidation products (mean change = −8.32; 95%

CI −14.22 to −2.43) over time of follow-up was observed (Table 2).

**Table 2:** The effect of 12 weeks of Nordic Walking (NW) training on indicators of oxidative stress balance, lipid profile and iron metabolism

	Baseline	After 12 weeks of training	p
PON ase [U/l]	156.68 ± 94.36	154.97 ± 92.22	0.47
AREase [kU/l]	164.99 ± 51.55	162.86 ± 52.04	0.3
MDA [μmol/l]	2.13 ± 0.56	1.92 ± 0.50	**0.01**
FRAP [mmol/l]	1.41 ± 0.23	1.45 ± 0.24	0.06
AOPP [μmol/l]	66.27 ± 27.08	57.95 ± 26.15	**0.01**
TC [mg/dl]	230.69 ± 46.89	231.31 ± 41.56	0.22
HDL [mg/dl]	70.77 ± 19.26	75.29 ± 21.11	**0.00**
LDL [mg/dl]	137.86 ± 43.54	133.91 ± 39.34	0.97
TGL [mg/dl]	110.26 ± 43.11	111.11 ± 43.91	0.39
Fe [µg/dl]	108.29 ± 32.76	89.43 ± 24.48	**0.00**
Ferritin [ng/ml]	99.36 ± 62.69	81.43 ± 61.67	**0.00**
Glucose [mg/dl]	97.86 ± 14.47	95.54 ± 12.12	0.2

Values are means (±SD)

*PONase* activity of paraoxonase-1 towards paraoxon, *AREase* activity of paraoxonase-1 towards phenyl acetate arylesterase, *MDA* malondialdehyde, *FRAP* ferric reducing ability of plasma, *AOPP* advanced oxidation protein products, *TC* total cholesterol, *HDL* high density lipoprotein, *LDL* low density lipoprotein, *TGL* triglycerides, *Fe* blood iron

### Lipid profile

Analyses of lipid profile show the only change in high-density lipoprotein which was significantly higher after the training (mean change = 4.51; 95% CI 1.47–7.56) (Table 2).

### Iron metabolism

The applied training programme were found to have caused a significant change in iron metabolism. A significant decrease in blood iron and ferritin concentration was observed. In addition, we discovered a significant correlation between glucose and ferritin level before (r = 0.46, p = 0.006) and after (r = 0.53, p = 0.001) the training period. Further analysis also revealed that the impact of training on the level of AOPP is correlated with the level of ferritin (r = 0.38, p = 0.03).

### Level of physical activity

All the components of physical fitness improved, however statistically significant differences were obtained in three of them: the endurance test: Chair stand (mean change = 2; 95% CI 1–3), 2-min Step (mean change = 14; 95% CI 5–23); and the flexibility test: Chair Sit-&-Reach (mean change = 7; 95% CI –5 to 9) (Table 3). Interestingly, a negative correlation between the blood ferritin and endurance test was recorded (r = −0.34, p = 0.03).

**Table 3:** Level of physical fitness

	Baseline	After 12 weeks of training	p
Chair stand (no. of stands)	21 ± 4	23 ± 5	**0.00**
Arm curl (no. of reps)	29 ± 4	30 ± 4	0.22
2-min step (metres)	145 ± 17	158 ± 26	**0.00**
Chair sit-&-reach (cm ±)	4 ± 11	11 ± 9	**0.00**
Back scratch (cm ±)	0 ± 7	−1 ± 8	0.92
8-foot up-&-go (s)	3.65 ± 0.7	3.44 ± 0.6	0.07

## Discussion

In the present study we demonstrate that the 12 weeks of Nordic Walking training significantly reduced levels of oxidative stress and this was accompanied by the drop in body iron stores. It is worth noting that the 12-week training decreased iron levels similarly to regular classes repeated for 32 weeks (Kortas et al. 2015). Iron is an essential metal participating in almost every process in living cells, including respiration, DNA synthesis, collagen synthesis and many others. Conversely, iron, due to its ability to lose and gain electrons, participates in redox reaction and formation of ROS. Therefore, iron metabolism must be strictly controlled as an increase in free iron—also called LIP—leads to augmented formation of iron-dependent ROS (Kruszewski 2003). Conversely, an increased LIP level leads to increased ferritin biosynthesis, which is an adaptive response, and consequently provides a subsequent drop in LIP.

Ferritin is an iron storage protein and, what is important, protects iron from redox reactions. Thus ferritin iron is considered to be safe protein; however, it has been demonstrated that during stress conditions activating protein kinases, ferritin undergoes degradation. This process is accompanied by ferritin iron release and an increase in iron-dependent ROS formation (Antosiewicz et al. 2006, 2007). Thus it can be speculated that ROS formation in stress conditions will be determined by the amount of iron stored in the ferritin. The obtained results revealed that NW training significantly reduced blood ferritin concentration. A previous paper demonstrated that blood ferritin correlates with body iron storage (Beutler et al. 2002), therefore it can be concluded that NW training reduces body iron stores. It has been documented that single exercise induces an increase in blood hepcidin (Antosiewicz et al. 2013). Thus it is possible that regular exercise results in lower iron absorption, which in consequence will lead to smaller iron stores. This data confirms the earlier observation that recreational exercise reduced iron stores in elderly subjects (Kortas et al. 2015). Interestingly, markers of oxidative stress are also diminished in the blood after 12 weeks of training. It can be speculated that lowering iron stores leads to less iron-dependent ROS formation. The data obtained on cell culture, that higher iron stores leads to higher ROS formation, support this conclusion (Warren et al. 1993). In addition to lower ferritin level, blood iron also drops significantly after training. However, a study performed on animals demonstrated that iron storage, rather than serum iron levels, contributed to the degree of oxidative stress (Noguchi-Sasaki et al. 2016).

There are several clinical data demonstrating that higher iron stores is associated with higher risk of a disease. For example, a strong correlation between blood ferritin and markers of metabolic syndrome has been observed in both men and women (Jehn et al. 2004). In our study a significant correlation between blood ferritin and fasting glucose level was noted. In our opinion, these data confirmed that exercise-induced lowering of body iron stores (blood ferritin) should be considered as a positive effect of the training. We did not assess any insulin sensitivity, but previously it has been demonstrated that training reduced insulin resistance (Slentz et al. 2011; Zoladz et al. 2008). Moreover, phlebotomy, which is considered to be the most effective means of iron withdrawal, has been shown to increase insulin sensitivity in type II diabetic patients (Fernandez-Real et al. 2002).

As mentioned earlier, higher intracellular iron leads to increased ferritin biosynthesis. Thus the effects of high iron stores may be mediated by iron or ferritin by itself. A heavy chain of ferritin (ferritin H) has been demonstrated to have some inhibitory effects on signalling pathways in which insulin is involved (Sengupta et al. 2009). Thus, lowering body iron stores of intracellular ferritin H protein concentration is expected to be downregulated too, which may ameliorate the insulin signalling pathway. Furthermore, high blood ferritin was also associated with a higher risk of heart disease. It was estimated that for each 1% increase in serum ferritin there was a more than 4% increase in the risk of heart attack in men. The risks further increase when accompanied by high blood cholesterol (Salonen et al. 1992). In our subjects no change in cholesterol was observed, however HDL cholesterol increased significantly.

## Conclusions

**Fig. 1:**
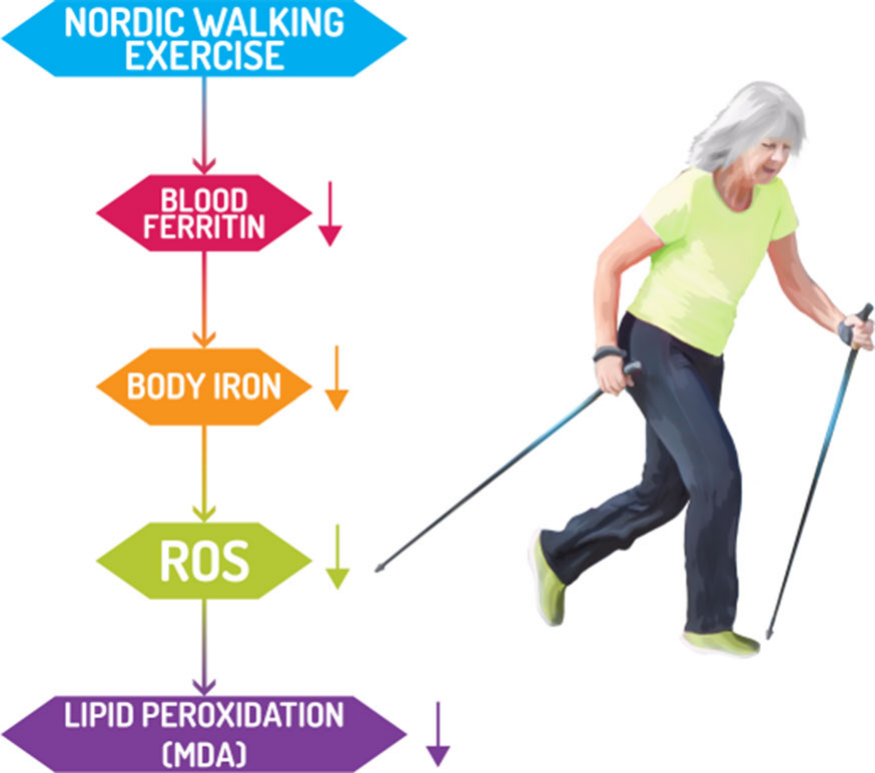
Nordic walking training decreased blood ferritin which is a good marker of body iron stores. Low iron is associated with both reduced iron-dependent ROS formation and lipid peroxidation

Based on previously published studies which demonstrated the beneficial effects of NW training (Lapszo et al. 2012; Latosik et al. 2014), the authors chose this kind of exercise as being the most appropriate for elderly subjects. We also observed that the applied training significantly improved fitness, measured by specific exercise tests, and a significant correlation between an improvement in physical performance and a drop in blood ferritin was observed. These data are consistent with a previous study performed on young men which showed that high blood ferritin was associated with a higher risk of low cardiovascular fitness (Mainous and Diaz 2009). To the best of our knowledge this is the first published report demonstrating that stored iron correlates with oxidative stress, fasting glucose level and physical fitness in elderly subjects (Fig. 1).
